# Innovations to Improve Lung Isolation Training for Thoracic Anesthesia: A Narrative Review

**DOI:** 10.3390/jcm13071848

**Published:** 2024-03-23

**Authors:** Corinne Grandjean, Gabriele Casso, Leslie Noirez, Manuel Granell Gil, Georges L. Savoldelli, Patrick Schoettker

**Affiliations:** 1 Anesthesiology Department, Hospital of Fribourg, 1708 Fribourg, Switzerland; corinne.grandjean@h-fr.ch; 2 Anesthesiology and Intensive Care Department, Istituto Cardiocentro EOC, 6900 Lugano, Switzerland; gabriele.casso@eoc.ch; 3 Pneumology Department, University Hospital Lausanne, 1005 Lausanne, Switzerland; leslie.noirez@chuv.ch; 4 Anesthesiology Department, University of Valencia, 46010 València, Spain; manuel.granell@uv.es; 5 Division of Anaesthesia, Department of Anaesthesiology, Clinical Pharmacology, Intensive Care and Emergency Medicine, Geneva University Hospitals, Faculty of Medicine, University of Geneva, 1205 Geneva, Switzerland; georges.savoldelli@hcuge.ch; 6 Anesthesiology Department, University Hospital Lausanne, 1005 Lausanne, Switzerland

**Keywords:** virtual reality, endoscopy, simulation, training, lung isolation, separation, thoracic anesthesia

## Abstract

A double-lumen tube or bronchial blocker positioning using flexible bronchoscopy for lung isolation and one-lung ventilation requires specific technical competencies. Training to acquire and retain such skills remains a challenge in thoracic anesthesia. Recent technological and innovative developments in the field of simulation have opened up exciting new horizons and possibilities. In this narrative review, we examine the latest development of existing training modalities while investigating, in particular, the use of emergent techniques such as virtual reality bronchoscopy simulation, virtual airway endoscopy, or the preoperative 3D printing of airways. The goal of this article is, therefore, to summarize the role of existing and future applications of training models/simulators and virtual reality simulators for training flexible bronchoscopy and lung isolation for thoracic anesthesia.

## 1. Introduction

Thoracic anesthesia is most commonly performed by skilled anesthetists (specialists, board-certified, senior attending) [1]. Nevertheless, flexible bronchoscopy (FB) and lung isolation (LI) to provide one-lung ventilation (OLV) remain complex procedures that may have a dramatic clinical impact in case of problems. LI can be achieved using three different devices: double-lumen tubes (DLTs), bronchial blockers (BBs), and single lumen tubes (rarely used at present) [2]. Strictly speaking, “isolation” of the lung implies functional sealing and can be achieved with BB or a DLT, while “separation” of the lungs refers to complete “anatomical” sealing and is achieved with a DLT [2]. Device malposition after insertion is the most frequent issue [3] as it has been demonstrated that inexperienced practitioners take two to three times longer than experts to place a device, and their positioning failure rates approach 40% [4]. Moreover, patients scheduled for surgeries needing OLV are often fragile with complex pathologies.

In this context, proper identification and understanding of the anatomy, training in the procedure, and planning are important aspects to ensure the success of the procedure. LI requires specific competencies such as accurate tracheobronchial anatomical knowledge [3,4], 3D vision, technical skills, preparation and planning, strategical thinking, and teamwork skills. Simulation can play a crucial role in procedural training to improve the management of LI and OLV. It provides an increasing realistic environment to practice technical and non-technical skills, ensuring competence and confidence in performing procedures critical for patient safety [5,6].

The field related to teaching and understanding the procedure of LI in thoracic anesthesia, and to the number of devices available to train people in it, is evolving rapidly. This work aims to review the current existing training models and virtual reality simulators and to summarize their role and impact on teaching and learning FB and LI for thoracic anesthesia.

## 2. Materials and Methods

A literature search was performed using Pubmed and Google Scholar databases for articles published in English between 2013 and 2023 with keywords related to thoracic anesthesia, OLV, fibroscopy and flexible bronchoscopy (FB), simulation, training, and virtual reality (VR). The initial search retrieved a substantial number of articles unrelated to this literature survey. We also searched reference lists from selected articles to identify additional relevant studies for this review.

## 3. Role of Flexible Bronchoscopy

Flexible bronchoscopy (FB) is of key importance in thoracic anesthesia. Its role is essential in both perioperative airway examination and LI and OLV techniques (the placement and control of specific devices such as DLTs and BBs). FB is an invasive medical procedure that requires technical skills, hand–eye coordination, and regular training [7,8,9]. Bronchoscopy complications are directly dependent, in an inverse relationship, on the operator’s experience: the less experienced, the more likely to encounter complications [10,11]. Furthermore, the overall level of FB expertise has been shown to decrease with the widespread practice of videolaryngoscopy by anesthetists for difficult airway management [12,13]. This gap needs to be filled by a different type of practitioner’s experience achievable through simulation training. Moreover, bronchoscopy remains, from a patient’s point of view, invasive and discomfortable [14]. Psychological safety provided by a simulated environment is therefore essential for the practitioner. Bronchoscopy simulations have become indispensable and have proved to be highly effective for educating anesthesiology residents [15,16].

## 4. The Ways of Teaching and Training

Traditional medical training, relying on lectures, workshops, and direct patient procedures, faces challenges like patient safety concerns, increased trainee numbers, and limited exposure to procedures. Simulation and virtual reality, proven effective in aerospace training, now play a crucial role in medical education, offering controlled, risk-free environments for practicing invasive techniques [17]. This shift allows for continuous learning, supported by mentorship and ongoing education on advancements in techniques.

## 5. Overview of Simulation Modalities, from Simple to Sophisticated

The field of medical simulation has been steadily expanding for many years, and at present, different types of simulators are available (Table 1). They offer a controlled environment where practitioners can repeatedly practice and refine their skills without the potential risks associated with live procedures [17].

Fidelity in clinical simulation is defined by the degree of realism, which is produced through the equipment, environment, and scenario [18]. Low-fidelity patient simulators are inanimate models from simple task trainers to anatomical models, dedicated to basic skill acquisition and coordination. High-fidelity patient simulators are usually computerized, contain hydraulics and compressors in order to mimic physiological responses, and have external monitors which display various somatic functions, allowing comprehensive training in complex situations.

Augmented reality (AR) refers to the integration of computer-generated information, typically visual or auditory, with the real-world environment to enhance medical education and training. AR overlays digital information onto the physical world [18], such as the additional identification of anatomical structures, allowing for a better understanding and an interactive learning experience.

Virtual reality refers to an immersive computer-generated simulation of a three-dimensional environment that can be interacted with in a seemingly real or physical way by individuals using electronic devices, with realistic sensory experiences, including sight, sound, and haptic touch, therefore offering an advanced training experience and facilitating effective learning [19]. VR enables the creation and continuous presentation of evolving clinical scenarios and has the added benefit of providing constantly changing tasks to maintain learner engagement and provide unpredictable new challenges and knowledge. In addition to teaching, VR can be used for competency assessment and training, especially for situations that occur infrequently in real life.

Immersive virtual reality (IVR), or sometimes also called alternate reality, is a term used to describe a completely fictional world that is different from our own reality, as an integral immersion into a learning life-like environment allowing a higher focus and emotional engagement [18].

This increasing offer of different simulators contributes to the ongoing advancement of simulation training in healthcare in general, and of course in thoracic anesthesia, providing diverse options to suit different learning preferences and needs.

## 6. Overview of Specific Simulators for Flexible Bronchoscopy and One-Lung Isolation Training

Simulation training for FB and LI in thoracic anesthesia focuses on hands-on and realistic experiences to enhance skills. The simulators currently available are classified according to their type in Table 2 and described below in this chapter. The prices of medical devices can vary greatly depending on the country and equipment supplier. To provide some guidance, costs are categorized as follows: prices under USD 1000 are considered low, prices between USD 1000 and 10,000 are considered moderate, prices between USD 10,000 and 30,000 are considered high, and prices over USD 30,000 are considered very high.

### 6.1. Wet Lab Simulation

Wet lab simulation means training on animal models, classically pigs, due to the relatively similar anatomy, using a real fiberscope. Live animal models are the most realistic endoscopy simulators: the haptic (tactile) feedback is identical to human tissue, although the thickness and orientation of various organs can be different. Additionally, secretions, respiratory motion, and bleeding with interventions replicate conditions encountered in clinical endoscopy. Ethical concerns about animal models for training, infrastructure requirements, and expense plead against this method of simulation, which is now reserved for more invasive procedures like tracheostomy [20,21].

### 6.2. Low-Fidelity Non-Anatomical Models

Low-fidelity simulation means the use of inanimate airway models into which real bronchoscopes can be inserted. There are a range of models, from simple task trainers to more modern simulators. Non-anatomical labyrinth models provide training in the movement of the wrist and hands, allowing basic skill improvement and familiarity [20]. Examples are the choose-the-hole model Cole Box^TM^ (University of Toronto, Toronto, ON, Canada), the Oxford Fiberoptic Teaching Box^TM^ (Pharmabotics Ltd., Nyewood, UK), and the modular training system Dexter^TM^ Endoscopic Dexterity Trainer (Replicant ^TM^ Medical Simulator Ltd., Wellington, New Zealand) that is composed of a series of channels and images. The limitations of such models are the lack of an incentive for realism and the risk of damage to the bronchoscpic equipment. However, training on both low- or high-fidelity models helps in improving procedural skills for fiberoptic intubation [22], and creative local low-fidelity models remain very affordable solutions to gain dexterity and hand–eye coordination.

### 6.3. Anatomical Models and Manikins

Anatomical models are detailed, life-sized representations of the human respiratory system, including the head, larynx, trachea, and bronchial tree, aiming to closely mimic the real structures. They are made of silicone and plastic-based materials, that replicate the texture and feel of human tissue. The most up-to-date models in this category consist of a mannequin composed of a head, a larynx, a tracheobronchial tree, and a thorax, to which a panel is attached to visualize the procedure externally, like the Laerdal Airway Management Trainer^TM^ (Laerdal; Stavanger, Norway) and the Airway Larry^TM^ (Nasco; Fort Atkinson, WI, USA). There is also the CLA Broncho Boy^TM^ model (CLA; Coburg, Germany), TruCorp Airsim bronchi^TM^ (Trucorp; Lurgan, N. Ireland, UK), and Koken Bronchoscopy Training Model^TM^ (Koken Co., Bunkyo-ku Tokyo, Japan), which have a detailed tracheobronchial tree down to the level of the first segmental bronchi [20].

These simulators allow for a better understanding of normal anatomy and advanced procedural skills, and can easily be used for interdisciplinary complex scenarios and the testing of non-technical skills. They are often rather expensive and specifically designed to be realistic for beginners.

### 6.4. Three-Dimensional-Printed Tracheobronchial Models

Commercially available classical intubation manikins are often expensive and not so accurate in the anatomical fidelity of the lower airway; therefore, they are not ideal simulators to teach and learn bronchoscopy or LI techniques. Three-dimensional printing of the tracheobronchial tree could be an interesting alternative to build more realistic and financially affordable bronchoscopy simulators in order to promote physical preoperative training. The idea is to create a hybrid simulator from an existing manikin with a 3D-printed lower airway with anatomical fidelity. Evaluation of this simulation modality has shown a favorable qualitative comparison in terms of realism, ergonomics, and price [23]. Recently, a new prototype of dynamic 3D printing bronchoscopic simulator obtained by assembling 3D reconstructions of different pathological chest CT scans into a single model has emerged, offering realistic and efficient conditions for training and teaching, including multiple pathologies [24].

**Figure 1:**
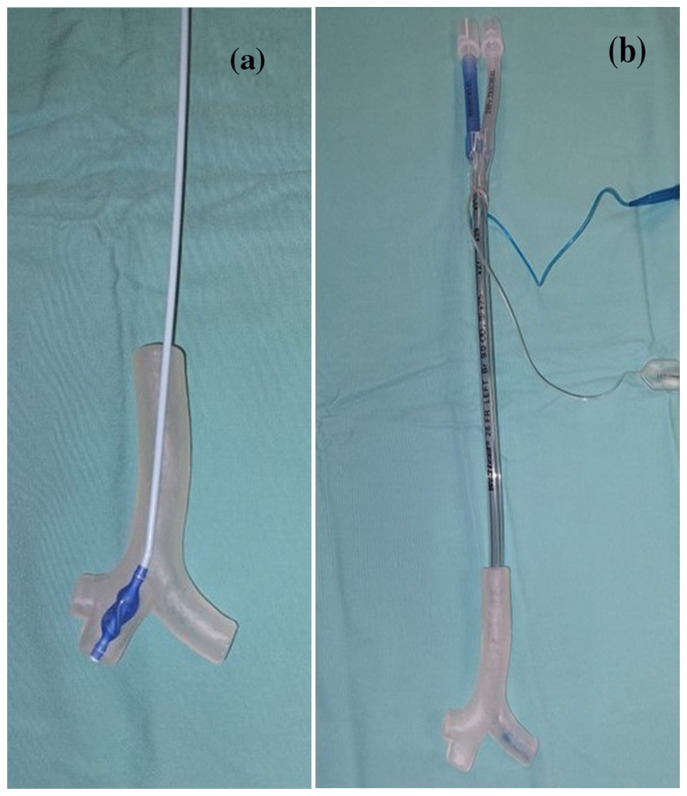
(**a**) Testing a bronchial blocker on a model. (**b**) Testing a double-lumen tube on a model. Reproduced with permission [26].

Three-dimensional-printed models promise to be of great value in the setting of airway pathologies [25], particularly in pediatrics. Three-dimensional modelling and subsequent printing or VR reconstruction are thus feasible for thoracic anesthesia cases. Such models can be used routinely in pediatric patients with challenging airway anatomy who are scheduled for thoracic surgery (Figure 1). They allow the selection and testing of the appropriate device and strategy for LI. In most cases, the result correlates well, in the case of high-quality imaging, with the final clinical finding [26].

Nevertheless, access to this promising technique requires a significant initial investment and a dedicated team, which is currently a barrier for many users. Complex models, such as airways, indeed demand design skills, experience, and time, as printing speed is reduced by the need for high resolution. Technically, the durability of sophisticated printed models can be an issue since flexible polymers are susceptible to weakening with force and time. For all these aspects, collaboration between industrial designers and healthcare practitioners should be encouraged [15].

### 6.5. Computer-Based Simulations

Computer-based simulations refer to web- or computer-based programs, software, or applications that can be run using a computer, tablet, or smartphone for self-learning without the need for other hardware devices.

For example, ThoracicAnesthesia.com is a free internet-based education, information, and reference service for thoracic surgery anesthesia issues that offers an online bronchoscopy simulator. Navigation is accomplished by clicking the arrows on the screen. The user can follow the path of the bronchoscope. If desired, labels can be added or removed from the map. Using real-time video, the simulator is designed to aid in the teaching and review of the complex bronchoscopic tracheobronchial anatomy. By improving their anatomical knowledge, anesthesiologists can become better performers of, and become more accurate in, intraoperative bronchoscopy during thoracic surgery.

As another example, the AURA study [27] has investigated the integration of a virtual reality gaming application into conventional didactic training for FB intubation for added self-directed learning. Their device allows users to practice virtual intubations with realistic visuals and controls using their mobile phones or tablet devices, while providing continuously evolving clinical scenarios integrating diverse and unpredictable practice tasks. The results were positive in terms of the accuracy of manipulation and confidence but non-significant in terms of time. Junior physicians appear to benefit more from such training compared to senior physicians.

Applications for smartphones regarding DLT placement and fiberoptic bronchoscopy are now available. For example, the Double Lumen^TM^ powered by Crystal Clear Solutions provides a teaching part containing video tutorials with AR and a simulator with scenarios, in order to improve anatomical and procedure visual knowledge. With regard to intubation technique, some authors have compared the effectiveness of training with an airway model simulator versus digital video disc (DVD)-based instructions in place of double-lumen endotracheal (DLT) tubes by anesthesiologists with limited thoracic experience [28]. Both groups performed better results than they achieved in their prior study [29], reducing the time to perform the correct lung isolation. Therefore, all these methods should be considered when training anesthesiologists to successfully place DLTs and perform FB procedures.

### 6.6. Virtual Reality Simulators

In professional training contexts, across multiple domains, notably in healthcare and formal education, it has been established that VR simulators significantly enhance motor skills, even if the specific contributions of some features, such as immersivity, fidelity, and interactivity, remain ambiguous [19]. In thoracic anesthesia, high-fidelity simulation for FB and LI techniques is based on the combination of fake “flexible scopes” combined with computers for the virtual projection of realistic airway models. This provides a lifelike environment, with virtual patients demonstrating realistic responses including vital signs and potential complications such as hypoxia and hypotension, as well as a simulation of awake sedation and topical anesthesia. These VR simulators allow gaining advanced anatomical knowledge of airways, safe training in the intentional sequencing of case topics and difficult pathological cases, automated scoring and objective feedback assessment, and unrestricted availability. A recent systematic review confirmed the findings of previous ones, indicating that using virtual reality bronchoscopy simulators for teaching and training represent an effective method, especially for novices, in terms of improving performance and reducing errors [30,31]. These VR simulators are often cumbersome machines, similar to pulmonologists’ bronchoscopes and video systems. They are very expensive and not easily transportable in the operating room (OR).

#### 6.6.1. Immersion AccuTouch ^TM^ Endoscopy Simulator

The PreOp Endoscopy Simulator^TM^ and its new versions AccuTouch ^TM^ Endoscopy Simulator and CAE EndoVR™ Interventional Simulator (HT Medical Systems; Rockville, MD, USA) consist of a simulated flexible scope and a computer with a monitor and software for simulating the procedure. The interface is a replica of the human face, with an access area in the nasal region for insertion of the scope, allowing both gastrointestinal, including ERCP, and bronchial procedures. This VR simulator offers learning modules for bronchoscopy, with patient cases developed using real patient data and physiological models. The software includes didactic content for tasks, covering training objectives, instructions, demos, and case histories for a customized curriculum to match learner needs. There is also hardware for detecting movements performed by the machine operator and capable haptic feedback to simulate the mechanical resistance of a real examination, as well as the coughing and respiratory movements of the patient. At the end of the examination, the equipment provides metrics related to the performance of the trainee, allowing one to track time, proficiency, dexterity, and complications for each task. Its high utility in training for FB in thoracic anesthesia has been well demonstrated [32].

#### 6.6.2. Simbionix BRONCH Mentor^TM^

Another VR simulator is the Simbionix BRONCH Mentor^TM^ (Surgical Science, Gothenburg, Sweden). It is designed to support both team and solo training sessions, completing patient management with moderate sedation dynamic complications and reactive virtual patients. It provides a didactic environment to enhance the learning curve, including skill-targeted tasks, procedural tasks, and aided/un-aided clinical cases, each followed by comprehensive performance feedback, and includes the standardized curriculum for essential endoscopic skills and diagnostic bronchoscopy, a highly structured training module co-developed with the American College of Chest Physicians.

### 6.7. Portable Virtual Reality Simulators

Recent developments in VR simulators are moving towards miniaturization, making them more portable and affordable while maintaining high performance. However, for many of them, haptic feedback remains exclusive to expensive VR simulators.

#### 6.7.1. BRONCH Express^TM^

This virtual reality trainer is a portable table-top version of the Simbionix BRONCH Mentor^TM^ simulator (Surgical Science, Göteborg, Sweden), packed in a suitcase. It offers hands-on training for essential bronchoscopic skills, diagnostic bronchoscopy, and essential EBUS-TBNA, focusing on the core competencies alongside the procedural and clinical know-how required to build bronchoscopic confidence and competence for pulmonologists.

#### 6.7.2. ORSIM™ Operative Room Simulation

The ORSIM™ system (Airway Simulation Limited, Auckland, New Zealand) was designed in 2006 in New Zealand by an anesthesiologist as a new solution to improve flexible bronchoscopy training. The ORSIM™ consists of three main parts: a replica bronchoscope, a desktop sensor, and a laptop with a software program. It is small, portable, can be stored within a hand-held case near the OR for regular use, and is easily transported to classrooms and training facilities. ORSIM™ includes a wide range of difficult upper and lower airway scenarios and specific modules for pediatric specialists and for pulmonologists. ORSIM™ has been proven to be valid and reliable to teach and assess basic and advanced bronchoscopy skills in a virtual environment and prepare practitioners for airway management in real life [33,34]. Recent scientific evidence confirms that through preprocedural training with this type of simulator, there is a significant improvement in speed and accuracy during real flexible bronchoscopy [35,36].

#### 6.7.3. Computer Airway Simulation System™ (CASS)

**Figure 2:**
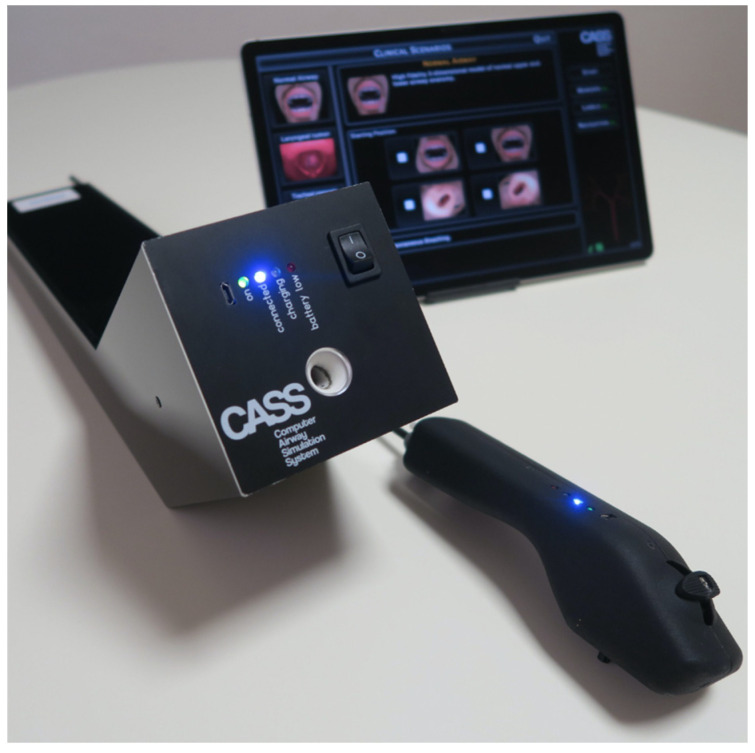
The Computer Airway Simulation System™.

The Computer Airway Simulation System™ (CASS) (Medvirt Ltd., Dino, Switzerland) is a new, affordable, ultra-portable VR bronchoscopy simulator recently developed in Switzerland [37]. This lightweight and completely wireless (Bluetooth) simulator consists of three elements: a realistic proxy bronchoscope including haptic feedback in case of collisions against the mucosa (which is a key feature, with 3D vision and interactivity, in facilitating effective learning [19]), a robotic patient interface, and a touch screen tablet (Figure 2).

**Figure 3:**
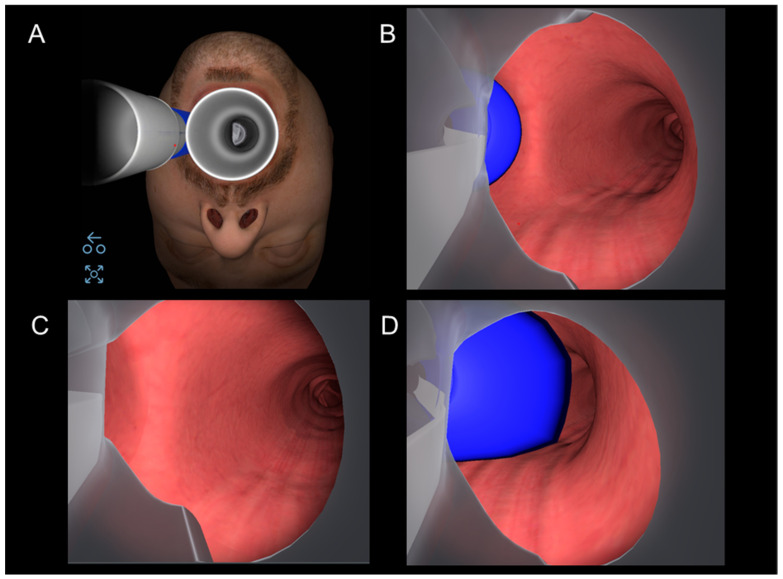
CASS™ double-lumen tube module. (**A**) Starting position; (**B**) DLT in correct position; (**C**) DLT too deep; (**D**) DLT too superficial with cuff herniation.

The CASS software includes different teaching modules and scenarios with normal and pathological three-dimensional anatomical models of the upper airway and tracheobronchial tree. A specific section for bronchoscopic assessment of double-lumen tube (DLT) positioning was recently included, and it is currently the only VR bronchoscopy simulator that allows the targeted teaching of LI for thoracic anesthesia. Four distinct scenarios are included: a left DLT in the correct position, one inserted too deeply, one too superficially, and a left DLT incorrectly inserted into the right main bronchus. Trainees can perform bronchoscopic inspection through both the tracheal and bronchial lumens and inflate and deflate the cuff to check for correct positioning or herniation (Figure 3). A final self-control learning action is included through the randomized choice of the DLT position to be checked.

### 6.8. Immersive Virtual Reality Simulators

Immersive virtual reality (IVR) simulators are head-mounted display technologies that provide total immersion in a lifelike environment for an enhanced learning experience with increased concentration and emotional engagement. Some simulators are designed to include the real-world environment and add augmented and virtual reality to achieve a state of mixed reality. The positive effect of IVR simulators has been demonstrated for patient hypnosis or distraction, as well as during a bronchoscopy procedure with breathlessness, coughing, and anxiety post-FB that were significantly less severe in the interventional group [38]. For teaching and training, IVR was first used for emergency and resuscitation training scenarios [39] and is now also used for airway assessment. A recent study in bronchoscopy shows that IVR simulation training improves the quality of diagnostic bronchoscopy in a simulated scenario with distractions compared to conventional simulation-based training [40].

## 7. Future

### 7.1. Precision Medicine: Introduction of Virtual Bronchoscopy into Simulators

**Figure 4:**
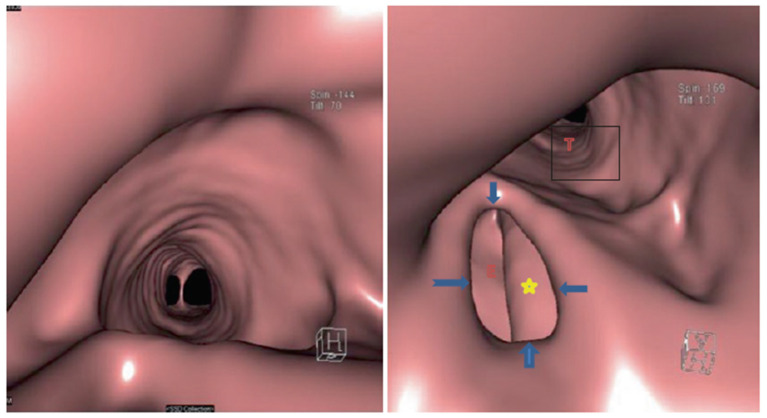
VB showing the trachea bifurcation (**left**), and on the (**right**), a tracheoesophageal fistula (TOF) is illustrated in detail. T: trachea, E: esophagus, ∗: NGT, and blue arrows show the TOF fistula’s boundaries. Reproduced with permission [43].

In parallel with the progress of virtual reality developments in advanced simulators, which is a fictional reality, imaging in radiology is advancing rapidly, both in terms of acquisition and processing. Virtual bronchoscopy (VB) is a newly emerging radiological technique that was first brought into clinical practice in 1993 [41,42]. At that time, CT imaging and computer software were not developed enough to produce a clinically relevant 3D model of the airways. Today, novel multi-detector computed tomography imaging techniques enable non-invasive intra- and extra-luminal evaluation of the tracheobronchial tree with minimal ionizing radiation. Furthermore, retrospectively generated virtual bronchoscopy from a routinely acquired computed tomography data set eliminates additional cost and radiation. Virtual bronchoscopy visualizes the inner structures through a perspective projection with an animated navigation of the inner structures of the bronchial tree made possible by computer-generated path tracking. This allows the clinician to obtain a realistic 3D reconstruction of the patient’s tracheobronchial pathology (Figure 4) and, thus, to plan the most appropriate strategy for performing the real bronchoscopy and/or LI technique. So far, the accuracy of tridimensional airway reconstruction is still highly dependent on CT imaging quality.

In the near future, VB will play a growing role in personalized medicine, starting with preoperative planning. Indeed, it can provide a 3D visualization of a patient’s unique airway anatomy, enabling the anticipation of challenges and selection of the most appropriate approach for LI and airway assessment as an accurate, non-invasive, and safe method, especially when lesions or deformities preclude conventional airway evaluation [34,43].

Artificial intelligence (AI) will soon make it possible to improve the quality and accelerate the creation of 3D reconstructions from thoracic CT-SCANs of patients with complex airway diseases. Integrating these reconstructions directly into a high-fidelity VR simulator would allow the physician to conduct bronchoscopy training in an ultra-realistic manner, preparing for all patient-specific difficulties and improving patient safety.

### 7.2. In Daily Routine

Previous studies have shown that a brief warm-up with a VR simulator increases the overall procedural performance, speed, and accuracy, even for experienced practitioners [35,36]. Given such benefits, VR simulators, in a portable format, are bound to become a new standard in clinical practice to be implemented in daily routine in thoracic anesthesia and other procedures requiring one-lung isolation techniques.

Real-time AI performance analysis will soon enable intraoperative guidance for the precise placement of LI devices based on the patient’s specific anatomy. Today, AI can anatomically interpret video bronchoscopy images of the carina and main bronchi, regardless of rotation or occlusion. The classification performance of AI models surpasses that of most human anesthesiology experts and is comparable to that of the most experienced pulmonologists [44]. Training with a novel AI algorithm allowed novices to perform more complete, systematic, and faster bronchoscopies on a mannequin in a recent study [45]. Case reports using FB with real-time virtual bronchoscopy navigation for procedures such as foreign body extraction [46] are beginning to appear in the literature.

### 7.3. Education and Curriculum

In education and training, AI developments will open the door to a multitude of settings and scenarios, allowing exposure and drills, even for experts (personalized training). The learning curve of virtual reality simulation seems to be 5 to 10 exposures to a specific airway scenario to reach fluency [47]. Skills and practice retention last for about two months [48] and can be maintained through additional simulation training, which is another advantage of the latter. Moreover, VR simulation can easily be integrated into immersive scenarios for interdisciplinary training. This adds a non-technical dimension of management to procedural skills, such as training for unexpected difficult intubation scenarios or acute hypoxemia during OLV in thoracic anesthesia [49]. Thanks to metric feedback, not only could VR simulation support alternative learning methods, but it could also provide factual data for evaluation; thus, it is likely to become part of future medicine assessment tools for university or post-graduate programs.

The transition from a case volume-based certification system to knowledge/skill acquisition and competency-based assessment is a current issue in medical education. This adaptation may be necessary due to the reduction in working hours and subsequent decrease in exposure to the clinical environment. Simulation-based mastery learning (SBML) is a form of competency-based training that has already been proposed as the next standard method for procedural task training in other endoscopic specialties, such as gastroenterology [50] and pulmonology [51].

The introduction of these helpful training methods into the curriculum for anesthesiologists in thoracic surgery is both relevant and desirable [52]. However, these methods are not yet explicitly suggested, except for TTE and TEE, in the second edition of the EACTAIC fellowship curriculum published in 2022 [53]. Virtual reality simulators are effective tools for supporting and disseminating a standardized and highly structured medical curriculum developed by a medical society. Some important societies, like the American Board of Thoracic Surgery (ABTS), recommend improving competencies in bronchoscopy for intensive care units, anesthesiology, thoracic surgery, and lung transplantation by performing all modalities of training, from simple online bronchoscope simulators, skill simulation and task training on plastic simulators, and simulation with virtual simulators to training on animal models and cadavers (in particular for invasive procedures), in order to enhance quantitative and qualitative learning experience [54].

In situations where high costs are an issue, it has been suggested that high-fidelity simulation should be offered in regional simulation centers, which should be accessible to all training programs [51].

## 8. Conclusions

Simulators, ranging from low- to high-fidelity devices, play a critical role in acquiring and maintaining the necessary skills for complex procedures, such as FB and LI in thoracic anesthesia. Continuous development in miniaturization, computer power, and artificial intelligence are opening up interesting new perspectives: personalized procedural planning associated with preoperative individualized realistic simulation training and real-time bronchoscopic guidance will be the next step to improve patient care and safety in thoracic anesthesia.

Simulation-based learning and training can meet the needs of multiple generations for various reasons and can support and disseminate a curriculum. While the educational benefits of simulation for FB and LI competencies are univocal, current disparate programs and assessment tools may not always provide clear evidence of their effectiveness from a patient’s perspective. However, younger physicians tend to appreciate and benefit from this new type of teaching and pedagogy. Improvements in technology and establishing dedicated education and assessment programs also promise an exciting future in the field of education in anesthesiology, with an opportunity to enhance the evaluation and measurement of skill transfer, directly benefitting patients and improving safety.

## Figures and Tables

**Table 1:** Simulation modalities and purposes.

Modality	Description	Distinct Purpose	Example
Low-Fidelity Simulation	Simple models	Basic skill acquisition and familiarity	Simple task trainers; Basic anatomical models for hands-on practice with equipment.
High-Fidelity Simulation	Realistic scenarios to replicate clinical conditions closely	Comprehensive training in complex situations	Advanced patient simulators that can mimic physiological responses
Augmented Reality (AR)	Integration of overlaying digital information onto the real-world environment	Enhancing the understanding of anatomical structures	Additional information during the placement of devices for one-lung isolation
Virtual Reality (VR)	Completely immersive, computer-generated environment	Enabling practitioners to navigate and practice one-lung isolation techniques in a realistic virtual setting	Virtual bronchoscopy simulators
Immersive Virtual Reality (IVR)	Fictional life-like setting that mimics the conditions encountered during a real procedure	Refine knowledge, skills, and decision making	VR headset technology

**Table 2:** Overview of simulators for FB and LI.

Modality	Examples	Cost
Wet lab simulation	Animal Models	Moderate
Low-fidelity models	Cole Box^TM^	Low Low
	Oxford Box^TM^ Dexter^TM^ Endoscopic Dexterity Trainer	Moderate
Anatomical models and manikins	CLA Broncho-Boy^TM^	Moderate
	TruCorp Airsim bronchi^TM^ Koken Bronchoscopy Training Model^TM^ Laerdal Airway Management Trainer^TM^ Nasco Advanced Airway Larry^TM^	Moderate Moderate Moderate Moderate
3D-printed tracheobronchial models	3D reconstruction software (e.g., OsiriX MD ^TM^) + 3D modelling software + 3D Printer (system in 3 parts)	Moderate
Computer-based simulations	Thoracicanesthesia.com (bronchoscopy simulation online) Airway Ex^TM^ App Double Lumen^TM^ App	Free Free Free-low
Virtual reality simulators	CAE AccuTouch Endoscopy Simulator^TM^ (previous PreOp^TM^) Simbionix BRONCH Mentor^TM^	Very high
Portable virtual reality simulators	BRONCH Express^TM^ ORSIM^TM^ Computer Airway Simulation System (CASS)^TM^	High High Moderate
Immersive virtual reality simulators	Immersive VR experience with headset technology Imersifi Bronchoscopy^TM^	Moderate

## Data Availability

Not applicable.
